# Retinoic acid signaling in growth plate development: receptor-specific effects, antagonist evidence, and pediatric translational considerations

**DOI:** 10.3389/fendo.2026.1962290

**Published:** 2026-10-06

**Authors:** Yutong Xing, Ruiying Fan, Kaixi Wang, Xinyu Ma, Fan Xuan, Huifeng Zhang

**Affiliations:** 1 Department of Paediatrics, The Second Hospital of Hebei Medical University, Shijiazhuang, Hebei, China; 2 Hebei University of Chinese Medicine, Shijiazhuang, Hebei, China

**Keywords:** chondrocyte, endochondral ossification, epiphyseal closure, growth plate, pediatric growth, RAR antagonist, retinoic acid, retinoic acid receptor

## Abstract

The growth plate is a spatially organized cartilage structure that drives longitudinal bone growth and is particularly vulnerable to nutritional imbalance, systemic disease, trauma, and drug exposure during childhood. Retinoic acid (RA), the bioactive metabolite of vitamin A, regulates transcription through retinoic acid receptors (RARα, RARβ, and RARγ). Its skeletal effects depend on concentration, developmental timing, tissue context, and receptor subtype. Physiologic RA homeostasis contributes to orderly chondrocyte maturation, whereas excessive or spatially inappropriate signaling can suppress proliferation, alter hypertrophic differentiation and extracellular matrix, and promote premature growth plate senescence or closure in experimental models. This narrative review integrates evidence on RA metabolism, RAR-dependent chondrocyte fate, and experimental RAR antagonists. Model-dependent antagonist effects include concurrent prevention of hedgehog inhibitor-associated closure and early post-injury attenuation of growth inhibition in juvenile mice; prevention during induction is not reversal of established physeal damage. Human zone-specific receptor biology remains insufficiently resolved, and apparently conflicting findings reflect differences in receptor pharmacology, tissue, developmental stage, timing, dose, and endpoint. Pediatric experience with systemic retinoids, including isotretinoin and the RARγ agonist palovarotene, underscores the developmental hazards of pathway modulation. Future studies should prioritize validated human growth plate models, receptor-subtype pharmacology, and longitudinal skeletal monitoring. RAR antagonism remains a preclinical, hypothesis-generating strategy requiring rigorous dose-window and long-term safety evaluation before pediatric clinical investigation.

## 1 Introduction

Longitudinal bone growth depends on coordinated proliferation, hypertrophy, matrix production, vascular invasion, and ossification within the epiphyseal growth plate. The resting, proliferative, prehypertrophic, and hypertrophic zones are biologically distinct but continuously connected, and disruption of their spatial organization may result in growth arrest, angular deformity, or reduced adult height (1–3). Human genetic data further indicate that genes enriched in growth plate chondrocytes contribute substantially to normal variation in stature, reinforcing the clinical importance of this tissue in pediatric growth disorders (4).

RA is generated from vitamin A through tightly regulated enzymatic steps and acts primarily through nuclear RARs that heterodimerize with retinoid X receptors (RXRs) (5–7). RA signaling is not simply beneficial or harmful. Its effect depends on local ligand availability, receptor expression, developmental stage, and interaction with pathways such as Wnt/β-catenin, bone morphogenetic protein (BMP), Indian hedgehog (Ihh), parathyroid hormone-related protein (PTHrP), and fibroblast growth factor (FGF). Nutritional vitamin A inadequacy may impair normal development, whereas hypervitaminosis A and pharmacologic retinoid exposure can adversely affect bone and cartilage (8).

This dose-sensitive biology is particularly relevant to children. Premature epiphyseal closure has been reported after systemic isotretinoin exposure, especially in pediatric oncology settings in which doses and cumulative exposure may exceed those used for acne (9–11). In the open-label MOVE program, palovarotene, a selective RARγ agonist developed for fibrodysplasia ossificans progressiva, was associated with a signal of activity against heterotopic ossification and with physeal toxicity in skeletally immature participants (12). Conversely, experimental RAR antagonists have attenuated growth plate closure or growth inhibition in selected mouse models (13, 14), illustrating the narrow margin between pathway modulation and developmental injury.

Systemic retinoid exposure is clinically relevant across selected pediatric and adolescent populations, although it is not universal. Isotretinoin is prescribed to adolescents with severe recalcitrant acne and is used under different schedules in selected pediatric oncology settings; all-trans retinoic acid (ATRA) remains essential therapy for acute promyelocytic leukemia; and palovarotene is used for defined pediatric skeletal indications in jurisdictions where it is approved. These populations differ in age, disease, cumulative exposure, concomitant therapy, and remaining growth potential, supporting proportionate skeletal-safety surveillance and better exposure–outcome evidence (9–12).

This review examines RA/RAR signaling as a context-dependent regulator of growth plate biology, distinguishes receptor agonism from antagonism, evaluates antagonist evidence by receptor subtype and model, and defines safeguards for pediatric translation.

## 2 Literature search strategy

A targeted narrative literature search was conducted in PubMed/MEDLINE from database inception through September 2, 2026, with a focused revision update addressing pediatric systemic retinoid exposure, clinical and epidemiological growth outcomes, RARα/β/γ zonal distribution, human growth plate organoids, and prevention versus post-injury intervention. Search terms combined concepts for retinoid biology (“retinoic acid”, retinoid, “retinoic acid receptor”, RAR, RARα, RARβ, and RARγ) with skeletal-development terms (“growth plate”, epiphyseal plate, chondrocyte, endochondral ossification, cartilage, and longitudinal bone growth). Additional searches addressed RAR antagonists, isotretinoin, ATRA, palovarotene, premature epiphyseal closure, pediatric skeletal safety, organoids, and microphysiologic models. Reference lists of relevant reviews and primary studies were hand-searched. English-language original studies, clinically informative case reports or case series, regulatory documents, and high-quality reviews were considered when directly relevant. This was a targeted narrative search, not a systematic review; no formal study-level risk-of-bias assessment or meta-analysis was performed. The reporting and organization of this narrative review were informed by principles described in SANRA; however, no formal SANRA scoring was performed (15).

## 3 Growth plate biology and pediatric relevance

### 3.1 Zonal organization and cellular progression

The resting zone contains slowly cycling chondrocytes, including PTHrP-expressing cells with stem-like properties that can generate columnar descendants. Proliferative chondrocytes divide and align along the longitudinal axis, after which prehypertrophic and hypertrophic cells enlarge, alter matrix composition, and coordinate vascular and osteogenic invasion (1, 16). Hypertrophic chondrocytes are not merely terminal matrix-producing cells; lineage studies indicate that a subset can contribute to osteoblast and marrow stromal populations. This plasticity complicates any attempt to manipulate a single differentiation step without affecting subsequent bone formation.

Growth plate activity is regulated by endocrine signals, local soluble factors, mechanical loading, and intrinsic senescence programs. The Ihh-PTHrP feedback loop delays premature hypertrophy, whereas BMP, FGF, Wnt, mechanistic target of rapamycin, and other pathways modulate proliferation and maturation (17, 18). Recent work also indicates that hedgehog activation can redirect resting-zone descendants toward osteogenic fates in a temporally restricted manner (19). RA/RAR signaling intersects with several of these pathways and should therefore be understood as part of a network rather than as an isolated linear switch.

### 3.2 Why growth plate effects require pediatric interpretation

A growth plate exposure that is tolerated after skeletal maturity may have permanent consequences in a growing child. The clinical effect depends on chronological age, bone age, pubertal stage, remaining growth potential, anatomical site, exposure duration, and whether injury is symmetric. A small focal lesion may produce an angular deformity, whereas diffuse or bilateral injury may reduce growth velocity and final height. Rodent physes do not reproduce human epiphyseal fusion exactly; consequently, changes in growth plate thickness or long-bone length in juvenile mice should not be translated directly into estimates of human height loss. **Figure 1** summarizes the growth plate zones in which RA availability and receptor activity may alter chondrocyte behavior.

**Figure 1:**
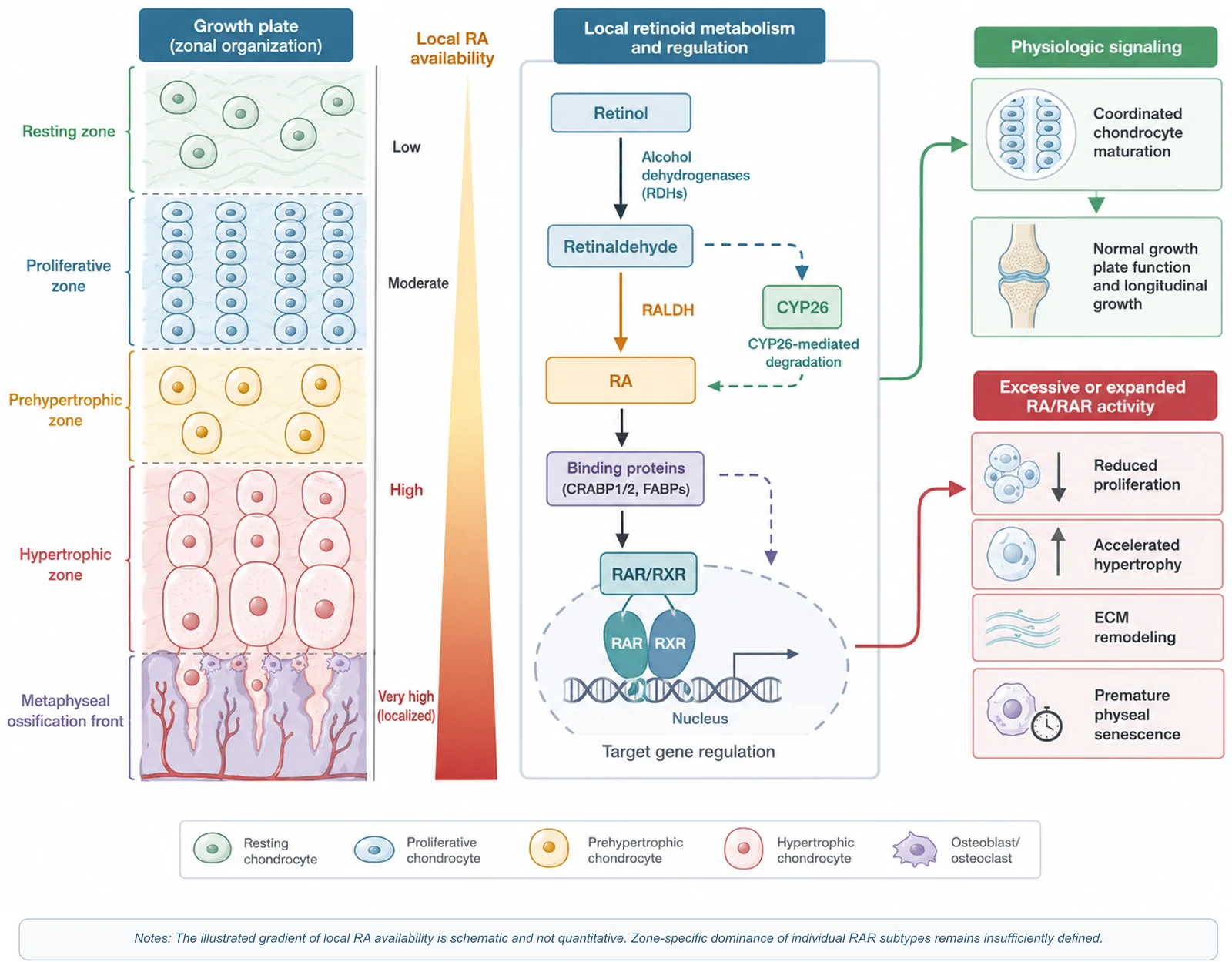
RA/RAR regulation across growth plate zones. Conceptual schematic. The figure depicts the resting, proliferative, prehypertrophic, and hypertrophic zones together with the adjacent metaphyseal ossification front. Local RA availability is determined by retinol/retinaldehyde metabolism, retinaldehyde dehydrogenases, CYP26-mediated degradation, binding proteins, and receptor expression. Physiologic signaling contributes to coordinated maturation, whereas excessive or spatially expanded RA/RAR activity may reduce proliferation, accelerate hypertrophy, alter matrix remodeling, and promote premature physeal senescence. The illustrated gradient of local RA availability is schematic and does not represent quantitatively measured RA concentrations across individual growth-plate zones. The pattern should not be directly extrapolated across species or developmental stages. Zone-specific dominance of individual RAR subtypes remains insufficiently defined and is therefore not assigned. CRABP1/2, cellular retinoic acid-binding proteins 1 and 2; CYP26, cytochrome P450 family 26; ECM, extracellular matrix; FABPs, fatty acid-binding proteins; RA, retinoic acid; RALDH, retinaldehyde dehydrogenase; RAR, retinoic acid receptor; RXR, retinoid X receptor.

## 4 Retinoic acid homeostasis in skeletal development

### 4.1 Synthesis, transport, and degradation

Retinol is oxidized to retinaldehyde and then to RA. Retinol dehydrogenase 10 (RDH10) contributes critically to the first oxidation step during embryogenesis, whereas retinaldehyde dehydrogenases generate RA from retinaldehyde (20, 21). Cellular retinol-binding proteins and cellular retinoic acid-binding proteins influence substrate delivery, intracellular trafficking, and access to nuclear receptors or catabolic enzymes. RA is degraded primarily by cytochrome P450 family 26 enzymes, creating local boundaries of signaling activity (5–7). Direct visualization of endogenous RA has confirmed that developmental exposure is spatially graded rather than uniform (22).

This distinction corrects a common oversimplification: cellular retinol-binding protein does not simply transport RA for degradation, and tissue RA concentration cannot be inferred from circulating vitamin A alone. Local synthesis, binding proteins, receptor abundance, and CYP26-mediated clearance collectively determine whether a chondrocyte experiences transcriptionally active RA.

### 4.2 Nuclear receptor signaling

RARα, RARβ, and RARγ bind DNA as heterodimers with RXRs. In the absence of ligand, RAR-RXR complexes can recruit corepressors; ligand binding changes the associated transcriptional machinery and modifies target-gene expression (5, 6). RARγ is highly relevant to cartilage and chondrogenesis, but receptor effects vary by cell type and developmental context. RXRs are obligatory partners in canonical RAR signaling.

## 5 RA/RAR signaling in chondrocyte proliferation, hypertrophy, apoptosis, and ECM remodeling

### 5.1 Proliferation and commitment to hypertrophy

Classical growth plate studies showed that exogenous RA can reduce chondrocyte proliferation and promote maturation, although the magnitude and direction of individual responses depend on dose, developmental stage, and culture conditions (23, 24). Postnatal mouse models with altered RAR function further demonstrate that balanced receptor activity is required for skeletal growth, matrix homeostasis, and normal growth plate organization (25). Thus, RA is not appropriately characterized as uniformly pro- or anti-chondrogenic.

During maturation, RA has been associated with increased alkaline phosphatase (ALP), collagen type X, and mineralization-related programs (26). It can cooperate with BMP signaling through Smad1 and Smad5, while Wnt/β-catenin interactions influence chondrocyte phenotype and matrix turnover (27, 28). Apparent discrepancies between studies often reflect differences in ligand concentration, receptor subtype, cell origin, and whether the experimental endpoint is proliferation, early chondrogenesis, terminal hypertrophy, or osteogenic conversion.

### 5.2 Apoptosis and mineralization

Terminal growth plate maturation involves calcium handling, matrix vesicle formation, and regulated cell survival. Annexin-mediated calcium influx has been linked to chondrocyte maturation and apoptosis (29). Excessive RA exposure can amplify stress responses and reduce viable proliferative chondrocyte pools, but apoptosis is not an invariant outcome of RA signaling. Claims about B-cell lymphoma 2, caspases, or oxidative stress should be restricted to the specific model and exposure conditions in which they were measured.

### 5.3 Extracellular matrix remodeling

RA/RAR signaling can reduce cartilage matrix synthesis and increase matrix-degrading programs through interactions with Wnt/β-catenin and inflammatory pathways (28). Excessive RA impaired proliferation and differentiation in human fetal palatal chondrocytes, illustrating that human developmental chondrocytes may also be vulnerable to supraphysiologic exposure (30). Nevertheless, palatal chondrocytes, articular chondrocytes, mesenchymal stromal cells, and growth plate chondrocytes are not interchangeable. Evidence from one compartment should be used as mechanistic context rather than direct proof of a physeal effect. **Table 1** summarizes the principal biological processes and their evidence base, and **Figure 2** presents an integrated network model.

**Table 1:** Effects of RA/RAR signaling relevant to growth plate chondrocyte biology.

Biological process	Main effect of RA	Key molecules/pathways	Evidence type	Potential pediatric relevance
Proliferation	Physiologic signaling supports orderly development; excessive RA commonly suppresses proliferation.	Cell-cycle regulators; Wnt/β-catenin; SOX9	Growth plate cultures; mouse models; human developmental chondrocytes (23, 25, 29, 30)	Loss of proliferative capacity may reduce growth velocity; exposure conditions are critical.
Hypertrophic differentiation	RA can accelerate terminal maturation and collagen X/ALP-associated programs.	RARs; BMP-Smad1/5; Ihh-PTHrP; RUNX2	Chondrocyte cultures and developmental models (24, 26, 27)	Premature maturation may narrow the growth plate and deplete remaining growth.
ECM remodeling	Excess or context-specific signaling can reduce matrix synthesis and increase degradative pathways.	COL2A1; aggrecan; MMPs; Wnt/β-catenin; NF-κB	Human and animal cartilage models (28, 30, 39)	Matrix effects differ across growth plate, articular cartilage, and MSC models.
Mineralization and osteogenic transition	RA may promote mineralization at late stages while inhibiting earlier osteogenic programs at high exposure.	ALP; annexins; BMP; Smad1/5; RUNX2	Growth plate chondrocytes; preosteoblast cultures (26, 27, 29, 37)	Stage-specific effects preclude a simple pro-osteogenic or anti-osteogenic label.
Apoptosis and cell stress	High or sustained exposure can increase apoptosis or stress responses in selected models.	Annexin-Ca2+; caspases; oxidative stress	Cell and animal studies (29–33)	The response is dose-, duration-, and tissue-dependent.
Growth plate senescence/closure	Excessive retinoid signaling can accelerate thinning, senescence, and closure.	RA gradients; RAR activity; ITGB2-YAP-Wnt/β-catenin axis (proposed in one experimental model; awaiting independent replication)	Guinea pig, rat, mouse, and pediatric exposure reports (9–11, 31–33)	Human risk is clinically important, but incidence and individual susceptibility remain uncertain.

ALP, alkaline phosphatase; BMP, bone morphogenetic protein; ECM, extracellular matrix; Ihh, Indian hedgehog; MSC, mesenchymal stromal cell; PTHrP, parathyroid hormone-related protein; RA, retinoic acid; RAR, retinoic acid receptor.

**Figure 2:**
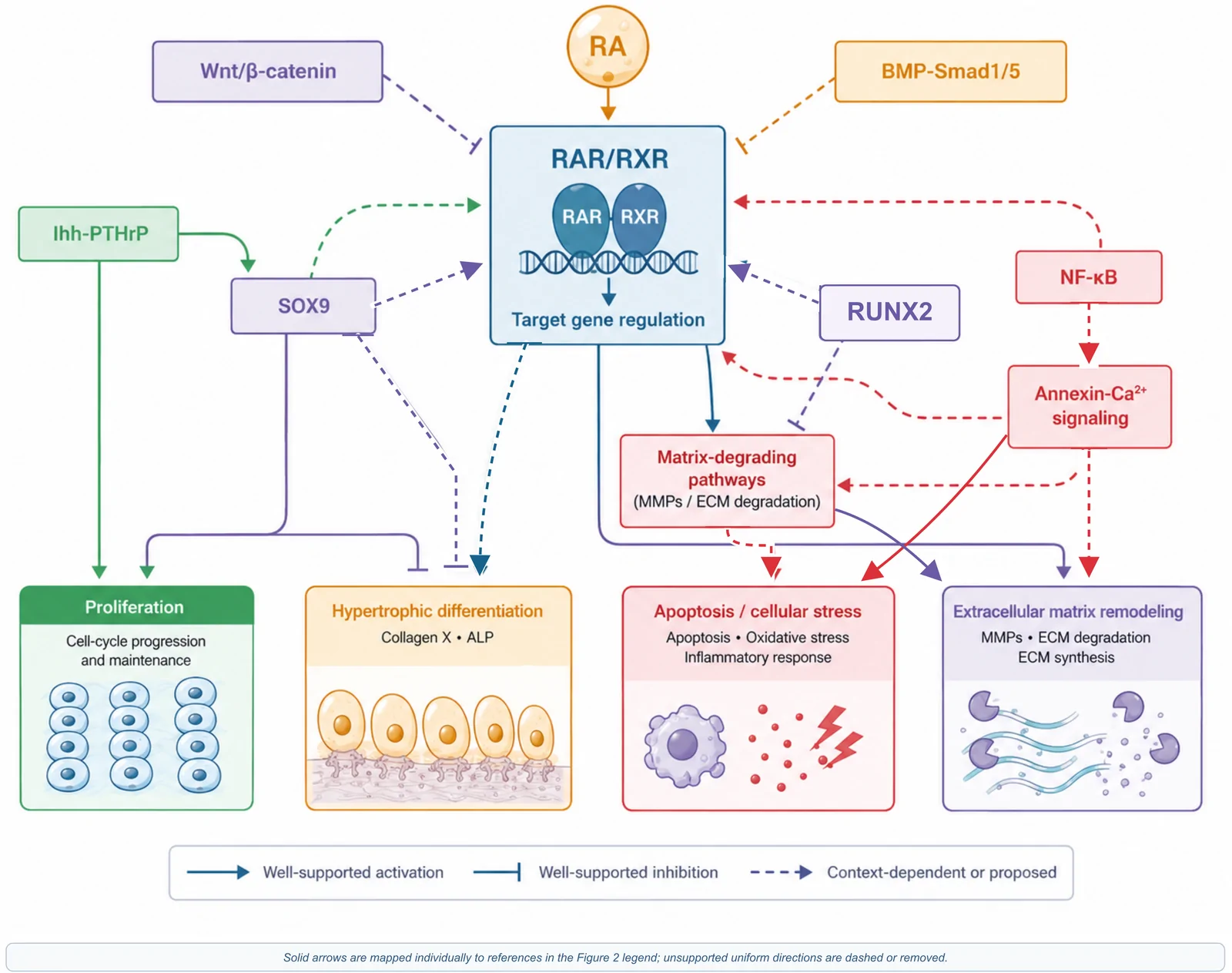
Molecular network of RA/RAR signaling in growth plate chondrocytes. Conceptual schematic. RA-bound RAR/RXR complexes regulate transcriptional programs that interact with Wnt/β-catenin, BMP-Smad1/5, Ihh-PTHrP, SOX9, RUNX2, NF-κB, annexin-calcium signaling, and matrix-degrading pathways. The schematic separates four outcomes: proliferation, hypertrophic differentiation, apoptosis or cellular stress, and extracellular matrix remodeling. Solid arrows are restricted to directional relationships directly supported in the cited experimental systems; dashed arrows denote indirect, context-dependent, or proposed interactions. Arrow-level evidence mapping for every retained solid relationship is: RA → RAR/RXR transcriptional activation (5, 6); Ihh-PTHrP → proliferation (3, 13, 17, 18); the PTHrP → SOX9 component (52); SOX9 → columnar proliferation (53); RAR/RXR → matrix-degrading pathways (28, 39); RAR/RXR → extracellular-matrix remodeling (23, 28, 39); annexin-Ca2+ signaling → apoptosis/cellular stress (29); and matrix-degrading pathways → extracellular-matrix remodeling (28, 39). Direct support was insufficient for a uniform directional relationship from SOX9 or RUNX2 to RAR/RXR, from RAR/RXR to hypertrophic differentiation across models, from NF-κB to annexin-Ca2+ signaling, from annexin-Ca2+ signaling to extracellular-matrix remodeling, or from matrix degradation to apoptosis; these paths are therefore dashed/context-dependent. Wnt/RAR and BMP/RAR interactions are also shown as context-dependent because their direction varies with ligand, cell type, and experimental stage (27, 28). These relationships should not be interpreted as universal across species, doses, or developmental stages. BMP, bone morphogenetic protein; ECM, extracellular matrix; Ihh, Indian hedgehog; MMP, matrix metalloproteinase; NF-κB, nuclear factor kappa B; PTHrP, parathyroid hormone-related protein; RA, retinoic acid; RAR, retinoic acid receptor; RXR, retinoid X receptor.

A recent experimental study from members of the present author group proposed an ITGB2-YAP-Wnt/β-catenin axis in ATRA-associated growth plate closure in rat and chondrocyte models (31). The present review does not reuse those experimental data and considers this pathway as one model within a broader receptor- and context-dependent evidence base. This proposed axis awaits independent replication.

## 6 Dose-, timing-, tissue-, and receptor subtype-dependent effects of RA signaling

### 6.1 Dose and duration

Skeletal responses to retinoid exposure do not follow a single linear dose-response across models. Basal RA signaling is necessary for development, whereas prolonged or high-level exposure has been associated with accelerated maturation and loss of proliferative capacity. Guinea pig and young-rat studies independently support exposure-related physeal toxicity (32, 33). These models establish biological plausibility but do not define a safe pediatric threshold because human exposure, metabolism, skeletal maturity, and treatment duration differ substantially.

### 6.2 Developmental timing and tissue context

Embryonic patterning, postnatal growth plate maintenance, articular cartilage homeostasis, and fracture repair involve different cell populations and signaling environments. A compound that suppresses chondrogenesis in heterotopic ossification may impair an open physis. Conversely, an antagonist that delays hypertrophy after a specific injury may interfere with normal endochondral bone formation if administered at another stage. This context dependence explains why findings from osteoarthritis, osteoporosis, or ectopic bone models cannot be presented as direct evidence for pediatric growth plate therapy.

### 6.3 Receptor subtype dependence

RAR subtypes share response elements but differ in expression, ligand selectivity, and biological effect. RARα-directed antagonism has been studied mainly in cell differentiation and teratogenicity models; RARβ inhibition has produced complex and sometimes unfavorable effects on cartilage matrix formation; and RARγ modulation has the strongest experimental link to chondrogenesis, cartilage turnover, and growth plate injury. Subtype selectivity is therefore necessary but not sufficient for safety. Tissue distribution, pharmacokinetics, partial agonism, RXR interactions, and developmental stage all remain relevant.

Vitamin A status adds another layer of complexity. Deficiency and excess can both impair skeletal health, but they should not be described as producing an identical phenotype or as uniformly causing premature closure (8, 34). Deficiency may compromise normal development and matrix biology, whereas excess retinoid signaling is more directly linked to accelerated maturation and physeal toxicity in available experimental and clinical reports.

### 6.4 Limits of zone-specific receptor mapping

Current evidence is insufficient to establish a reproducible zone-specific dominance map for RARα, RARβ, and RARγ across the human growth plate. In the postnatal mouse growth plate, RARγ was reported to be the most strongly expressed RAR subtype, with prominent expression in the proliferative and prehypertrophic zones (25). However, this species- and developmental-stage-specific observation should not be interpreted as a human anatomical map. Available studies vary in species and developmental age and often use whole-tissue extracts, isolated cells, or mRNA assays. Transcript abundance does not establish protein localization or receptor activity, and selective pharmacologic responses cannot be equated with anatomical dominance. Protein-level localization in human physeal tissue remains sparse.

A reliable map will require human growth plate tissue sampled across developmental stages, spatial transcriptomics or zonally resolved single-cell RNA sequencing, receptor-specific immunohistochemistry or immunofluorescence, and perturbation studies that connect localization with function. **Figure 1** therefore does not assign RARα, RARβ, or RARγ dominance to individual zones; such assignments require independent validation.

## 7 RAR antagonists in growth plate regulation: evidence by receptor subtype

### 7.1 RARα antagonists

Early RARα antagonists were developed as pharmacologic tools to oppose selected RA-dependent transcriptional effects. Ro 41–5253 counteracted RA responses in cell systems, and related antagonism reduced retinoid-associated malformations in experimental teratogenicity models (35, 36). These studies established receptor-selective antagonism as a mechanistic strategy, not as evidence for treatment of pediatric growth disorders. ER-50891 partially rescued ATRA-inhibited osteoblastogenic differentiation *in vitro*, but the model addressed preosteoblasts rather than an intact growth plate (37).

### 7.2 RARβ antagonists

The RARβ antagonist LE135 did not induce chondrogenesis in human mesenchymal stromal cell pellets and reduced cartilage matrix deposition when combined with transforming growth factor β (38). This negative or mixed result is important: receptor antagonism cannot be assumed to preserve cartilage, and effects on early chondrogenesis may differ from effects on terminal hypertrophy.

### 7.3 RARγ antagonists

RARγ has the largest antagonist evidence base among RAR subtypes in cartilage-related models. In juvenile mice, CD2665 was co-administered with sonidegib during the two-day induction regimen and prevented subsequent growth plate involution, preserving proliferative and reserve-zone features (13). This was a concurrent or prophylactic intervention, not treatment of established closure. In the needle-injury model, 7C was started one day after injury and given daily for seven days; it attenuated bone-bridge formation and partially rescued injury-associated growth inhibition (14). This constitutes early post-injury intervention within an acute repair window, not reversal of a mature lesion. Both studies used specific injury mechanisms, short experimental windows, and nonclinical exposure.

RARγ inhibition also opposed inflammatory cartilage degradation in osteoarthritis models using LY2955303 (39). In a separate BMP-2 model, 7C-loaded nanoparticles were co-formulated with BMP-2 at implantation and enhanced ectopic endochondral bone formation (40). These experiments answer different questions from protection of an open physis. **Table 2** therefore separates the two 7C studies and records intervention timing and experimental intent. No RAR antagonist has established efficacy or safety for prevention or treatment of growth plate injury in children.

**Table 2:** RAR antagonists and evidence for growth plate or cartilage regulation.

Antagonist	Target receptor	Experimental model	Intervention timing/experimental intent	Main finding	Evidence type	Translational limitation
Ro 41-5253/early RARα antagonists	RARα	Cell-differentiation; limb-bud micromass and mouse teratogenicity models	Mechanistic; concurrent with RA exposure or developmental-model induction	Counteracted selected RA-dependent effects and reduced experimental malformations.	Mixed preclinical	Not tested as a growth-preserving therapy; systemic developmental effects remain possible (35, 36).
ER-50891	RARα	Preosteoblast culture with ATRA and BMP-2	Concurrent *in-vitro* mechanistic rescue	Partially rescued ATRA-inhibited metabolic activity, osteocalcin expression, and mineralization.	*In vitro*	Preosteoblast endpoint; no intact physis, juvenile pharmacokinetics, or safety data (37).
LE135	RARβ	Human bone marrow/adipose MSC pellet culture	Concurrent *in-vitro* mechanistic experiment	Did not induce chondrogenesis and reduced TGF-β-associated cartilage matrix deposition.	*In vitro*	Mixed/unfavorable cartilage effects argue against assuming class benefit (38).
CD2665	RARγ	Juvenile mice receiving sonidegib	Concurrent with sonidegib during the two-day induction regimen; prophylactic/concurrent	Prevented sonidegib-associated growth plate involution and related molecular changes.	Juvenile mouse *in vivo*	Does not test reversal of established injury; single mechanism; no pediatric efficacy or long-term multisystem safety data (13).
LY2955303	RARγ inhibitor	Human chondrocytes and rat osteoarthritis model	Mechanistic/disease-model intervention	Opposed RARγ/NF-κB-associated cartilage degradation and inflammation.	Mixed preclinical	Articular-cartilage/OA evidence is not direct growth plate evidence (39).
7C	RARγ	Juvenile mouse experimental growth plate injury	Started one day after injury and continued daily for seven days; early post-injury, not established-lesion treatment	Attenuated bone-bridge formation and partially rescued injury-associated bone-growth inhibition.	Juvenile mouse *in vivo*	Injury-specific and timing-dependent; no human efficacy; long-term multisystem safety unknown (14).
7C	RARγ	BMP-induced ectopic endochondral bone formation	Co-formulated locally with BMP-2 at implantation; concurrent/mechanistic	Enhanced BMP-induced ectopic endochondral bone formation.	Preclinical *in vitro* and mouse *in vivo*	The direction of effect differs from the growth-plate injury model, emphasizing strong model-, tissue-, and timing-dependence (40).

ATRA, all-trans retinoic acid; BMP, bone morphogenetic protein; MSC, mesenchymal stromal cell; NF-κB, nuclear factor kappa B; OA, osteoarthritis; RA, retinoic acid; RAR, retinoic acid receptor; TGF-β, transforming growth factor beta.

### 7.4 Reconciling conflicting and context-dependent findings

Apparently conflicting findings can arise without being mutually exclusive. Agonism and antagonism produce different transcriptional states, and RARα, RARβ, and RARγ cannot be treated as an interchangeable drug class. Experimental responses also depend on whether the target is a physiologic growth plate, an acutely injured physis, articular cartilage, a mesenchymal stromal cell, a preosteoblast, or a BMP-driven ectopic ossification site. Dose, duration, developmental stage, and the phase of endochondral differentiation being measured—early chondrogenesis, hypertrophic maturation, mineralization, or matrix degradation—can change both the direction and meaning of an endpoint. Preservation of an injured growth plate is not biologically equivalent to generalized inhibition of endochondral ossification. A beneficial effect in one disease model cannot be generalized to normal physeal growth or another skeletal context.

Intervention timing is especially important. Prevention of injury during model induction and treatment after an established growth plate lesion are biologically and translationally distinct scenarios. The CD2665 study tested concurrent prevention during sonidegib exposure; the 7C injury study tested an early intervention beginning one day after injury; and the 7C–BMP study tested concurrent mechanistic enhancement of bone induction. Evidence from concurrent administration should not be interpreted as proof that established physeal damage can be reversed. Future studies should prespecify whether the intent is prophylaxis, acute post-injury modulation, or treatment of an established lesion and should use endpoints appropriate to that intent.

## 8 Pediatric implications and clinical scenarios

### 8.1 Vitamin A deficiency and excess

Vitamin A is necessary for normal development, vision, immunity, and tissue differentiation. In children with restricted diets, malabsorption, liver disease, or other risk factors, deficiency should be diagnosed and treated according to established clinical guidance. However, supplementation beyond physiologic requirements is not a strategy to enhance growth. Excess preformed vitamin A and systemic retinoids can adversely affect bone turnover and skeletal development (8, 34). Clinical interpretation should therefore separate nutritional correction from pharmacologic manipulation of RAR signaling.

### 8.2 Isotretinoin and all-trans retinoic acid exposure

Human isotretinoin evidence should be interpreted hierarchically. A Rochester Epidemiology Project cohort compared 226 adolescents who initiated isotretinoin before age 15 with 1,179 acne controls treated with oral antibiotics; height velocity decreased transiently after initiation, but final height did not differ significantly (41). A nationwide Danish conscription-linked analysis of 379,196 individuals likewise found no clinically meaningful reduction in adult height after adolescent isotretinoin exposure, including analyses by age at initiation and cumulative dose (42). These studies improve population-level context but did not systematically image growth plates and remain susceptible to residual confounding and limited female or very-early-exposure subgroups. In contrast, a neuroblastoma series identified premature growth arrest in 3 of 216 exposed patients, but malignancy, cumulative high-dose exposure, chemotherapy, radiotherapy, endocrine dysfunction, and survivorship selection limit generalization of that estimate (10). Current isotretinoin labeling also records spontaneous reports of premature epiphyseal closure and adolescent bone-age changes; regulatory labeling is an important safety signal but is not an incidence study (43).

ATRA remains an essential component of pediatric acute promyelocytic leukemia treatment. A multicenter pediatric protocol study demonstrated the clinical importance of ATRA-containing therapy but was not designed to ascertain physeal injury, serial growth-plate imaging, or final height (44). Current prescribing information establishes pediatric use while emphasizing other acute retinoid toxicities (45). Potential skeletal toxicity should not discourage indicated therapy; it supports age-appropriate growth surveillance and survivorship follow-up, with explicit attention to cumulative exposure and disease- and treatment-related confounding. Available human evidence supports biological and clinical plausibility of retinoid-related physeal toxicity but does not currently permit reliable estimation of incidence, individual susceptibility, or a universally safe exposure threshold.

### 8.3 Palovarotene as a clinical cautionary example

Palovarotene is a selective RARγ agonist, not an antagonist. In the single-arm, open-label phase 3 MOVE program for fibrodysplasia ossificans progressiva, *post hoc* analyses suggested reduced new heterotopic ossification, while premature physeal closure or epiphyseal disorders were reported in 21 of 57 participants younger than 14 years (12). The rare-disease design, *post hoc* efficacy analyses, cumulative exposure, and disease-specific context limit generalizability (46, 47). Current U.S. prescribing information, revised March 2025, contains a boxed warning for premature epiphyseal closure and requires baseline skeletal-maturity assessment and continued growth and radiographic monitoring in growing pediatric patients (48). The label supports a safety signal and monitoring requirements; it should not be used as a population-level incidence study.

Palovarotene should not be presented as evidence that RAR antagonists are clinically effective. Its principal relevance to this review is safety: receptor-selective activity does not eliminate developmental toxicity, and a pharmacologic effect that is beneficial for pathological ossification may be harmful to a normal growth plate.

### 8.4 Drug-induced growth plate injury and monitoring

Potential drug-related physeal injury should be considered when a child receiving a retinoid or another pathway-targeted agent develops declining height velocity, disproportionate limb growth, persistent knee pain, angular deformity, or unexpected changes in bone age. Evaluation should be individualized and may include review of cumulative exposure, pubertal status, endocrine function, nutritional status, standing alignment, and targeted radiographs or magnetic resonance imaging. **Table 3** summarizes clinical risks, evidence gaps, and research priorities. The table is not a treatment guideline and does not support prophylactic antagonist use.

**Table 3:** Pediatric translational risks and future research priorities.

Clinical issue	Potential relevance of RA/RAR signaling	Key risk	Current evidence gap	Future research need
Vitamin A deficiency	RA is required for normal developmental signaling (5, 8).	Unnecessary high-dose supplementation; confounding by malnutrition and systemic disease.	Limited direct evidence linking deficiency to a specific human physeal phenotype.	Define nutritional status and separate replacement from pharmacologic pathway modulation.
Vitamin A excess/hypervitaminosis A	Excess retinoid exposure can alter bone turnover and chondrocyte maturation (8, 34).	Dose accumulation, multisystem toxicity, skeletal effects.	Individual susceptibility and pediatric exposure thresholds are uncertain.	Prospective exposure studies with growth, bone age, density, and fracture outcomes.
Isotretinoin or ATRA exposure	Case reports and oncology series support possible physeal injury, while recent acne cohorts did not show clinically meaningful final-height reduction (9–11, 41–45).	Irreversible focal or diffuse growth disturbance; disease and treatment confounding.	Physeal imaging was not systematic; incidence and susceptible windows remain poorly defined.	Prospective cohorts and survivorship registries with cumulative dose, standardized growth, and clinically indicated imaging.
Palovarotene (RARγ agonist)	The MOVE program showed a *post hoc* signal against heterotopic ossification and a clinically important physeal-toxicity signal (12, 46–48).	Premature epiphyseal closure, impaired growth, bone-density and reproductive risks.	Rare-disease, open-label/*post hoc* evidence limits generalizability.	Continue required growth/skeletal monitoring and long-term outcome registries.
Drug- or injury-induced growth plate damage	Excess local retinoid signaling may contribute in selected mouse models (13, 14).	Antagonism could disrupt normal repair or ossification.	Concurrent prevention and early post-injury intervention have not established reversal of mature lesions; no human efficacy data.	Human-derived models and staged studies that separate prophylaxis, acute post-injury intervention, and established-lesion treatment.
Experimental RAR antagonist strategies	May modify maturation in narrowly defined contexts (13, 14, 35–40).	Narrow therapeutic window, off-target developmental effects, unknown reproductive safety.	Cell and animal evidence only; no reliable pediatric dose threshold.	Subtype-specific dose-window studies and follow-up through skeletal maturity before any pediatric trial.

ATRA, all-trans retinoic acid; FOP, fibrodysplasia ossificans progressiva; RA, retinoic acid; RAR, retinoic acid receptor.

## 9 Translational barriers and safety concerns

### 9.1 Developmental toxicity and a narrow therapeutic window

Because RA signaling contributes to development across multiple organ systems, systemic RAR antagonism during growth carries theoretical developmental risks beyond cartilage, including effects on neural, immune, reproductive, and visual tissues (5, 6). A dose that attenuates excessive physeal maturation could also impair normal differentiation. The relevant exposure window may vary by age, sex, pubertal stage, growth plate site, and cause of injury. This narrow and dynamic dose-timing problem is a central obstacle to pediatric development.

### 9.2 Receptor selectivity, off-target effects, and pharmacology

Nominal receptor selectivity measured *in vitro* may not predict *in vivo* activity at clinical concentrations. Metabolites, partial agonism, tissue penetration, RXR availability, and transcriptional cofactor expression can change the functional response. Long-term studies must evaluate liver function, lipid metabolism, vision, neurobehavior, fertility, pregnancy risk, bone density, fracture, and effects on puberty in addition to longitudinal growth.

### 9.3 Species and model differences

Rodent growth plates persist differently from human physes, and experimental closure may not reproduce human epiphyseal fusion. Cell cultures lack vascular, mechanical, endocrine, and immune inputs. Osteoarthritis and ectopic ossification models answer different questions from growth plate protection. Accordingly, efficacy across at least two complementary models, including a human-derived platform, should precede any consideration of pediatric trials.

### 9.4 Current evidence-base limitations

The antagonist literature is small and heterogeneous. Studies differ in compound, receptor subtype, exposure schedule, injury mechanism, age, and endpoint; negative developmental findings may be underreported. No pediatric trial has established that an RAR antagonist preserves growth, prevents deformity, or improves adult height. **Figure 3** summarizes the current evidence and translational barriers.

**Figure 3:**
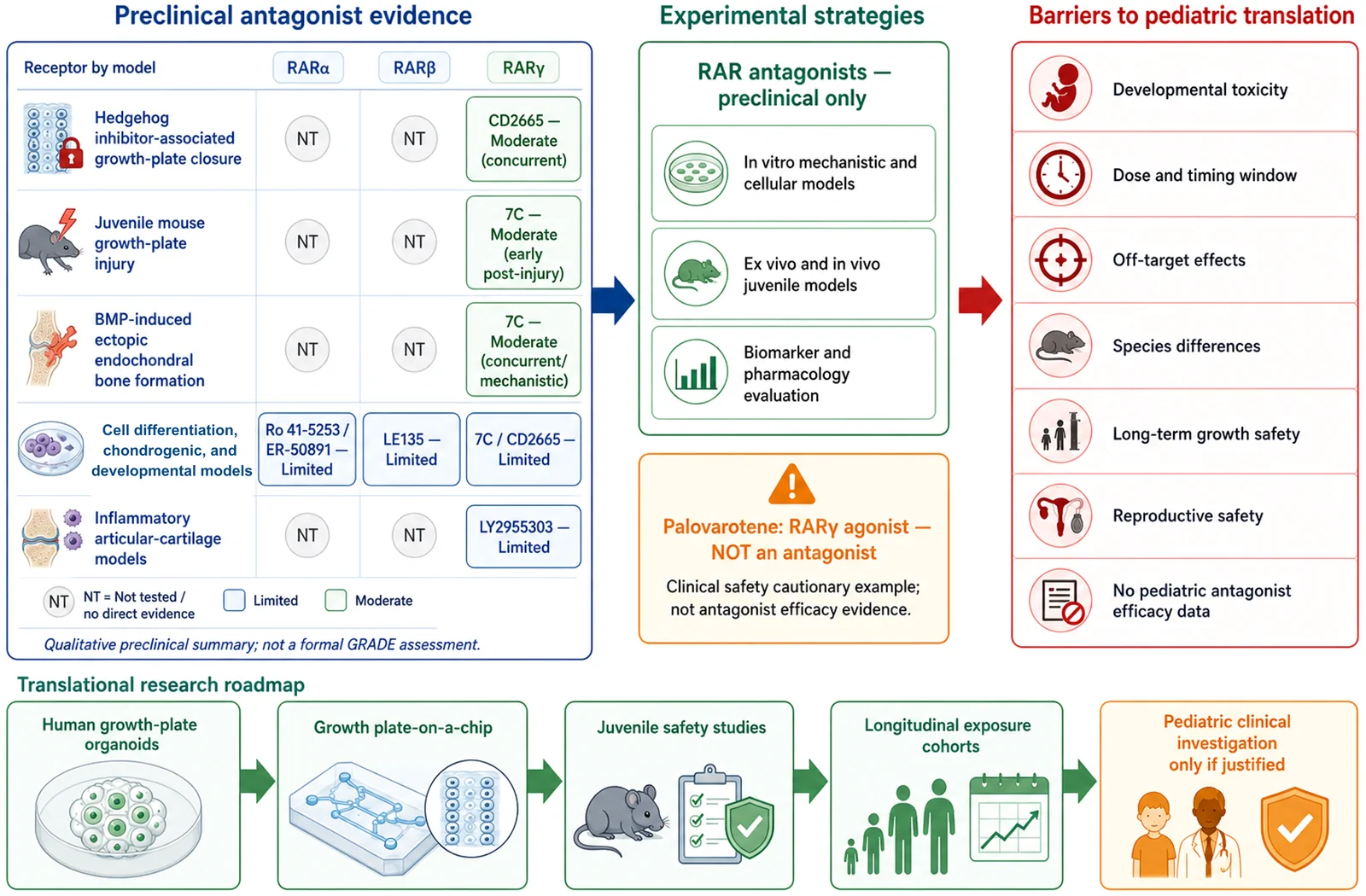
Preclinical RAR antagonist evidence and barriers to pediatric translation. Conceptual schematic. The receptor-by-model matrix distinguishes not tested/no direct evidence (NT) from Limited and Moderate qualitative preclinical evidence. These categories summarize the available literature and do not constitute a formal GRADE assessment. Direct model-compound mappings are: CD2665 for concurrent prevention in hedgehog inhibitor-associated closure (13); 7C for early post-injury intervention in juvenile growth plate injury (14); 7C for concurrent/mechanistic enhancement of BMP-induced ectopic endochondral bone formation (40); Ro 41–5253 and ER-50891 in RARα cellular or developmental models (35–37); LE135 in RARβ mesenchymal-stromal-cell chondrogenesis (38); 7C/CD2665 in selected chondrogenic assays (13, 40); and LY2955303 in inflammatory articular-cartilage models (39). NT does not mean low evidence; it means that no direct experiment was identified for that receptor–model combination. The mixed-model row is labeled ‘Cell differentiation, chondrogenic, and developmental models’ so that individual compounds are not implied to share the same assay type. Palovarotene is displayed as an RARγ agonist—NOT an antagonist—and serves only as a clinical safety cautionary example, not antagonist efficacy evidence. The roadmap proceeds from human growth plate organoids and growth plate-on-a-chip models through juvenile safety studies and longitudinal exposure cohorts; pediatric clinical investigation would require separate justification. BMP, bone morphogenetic protein; GRADE, Grading of Recommendations Assessment, Development and Evaluation; NT, not tested/no direct evidence; RAR, retinoic acid receptor.

## 10 Review-level methodological limitations

This narrative review used PubMed/MEDLINE as its single primary bibliographic database and was restricted to English-language literature. The search was targeted to the mechanistic and translational questions defined *a priori* and updated for reviewer-requested topics; it was not designed as an exhaustive systematic review. No formal study-level risk-of-bias instrument, certainty-of-evidence grading, or meta-analysis was applied. Study selection, grouping, and interpretation therefore retain an element of author judgment, and relevant reports indexed outside PubMed/MEDLINE may have been missed.

The evidence base itself is heterogeneous and potentially affected by publication bias. Regulatory documents, case reports, observational cohorts, cell studies, and animal experiments answer different questions and were not treated as equivalent evidence. Variation in species, developmental stage, exposure schedule, tissue, and endpoint also precluded quantitative aggregation. These limitations are consistent with the review’s aim: critical and translational synthesis rather than exhaustive quantitative evidence aggregation. Conservative language was used when direct human or zone-specific evidence was insufficient.

## 11 Future directions

### 11.1 Human experimental systems

Human pluripotent stem cells can model stages of skeletal development and provide a path toward patient-specific chondrocyte and osteogenic systems (49). Growth plate organoids should reproduce zonal architecture, ligand gradients, mechanical loading, vascular interfaces, and longitudinal transitions rather than merely express a panel of cartilage markers. Engineering a functional growth plate *in vitro* remains difficult (50), although a layered organoid model demonstrates progress toward spatially organized platforms (51). Current systems have imperfect resting, proliferative, and hypertrophic zonation; lack validated endogenous RA gradients and complete RALDH/CYP26 metabolic compartmentalization; and have uncertain fidelity of RARα, RARβ, and RARγ spatial expression. They incompletely reproduce the vascular–osteogenic interface, physiologic mechanical loading, GH–IGF-1, thyroid, and sex-steroid environments, and immune–stromal interactions. *In-vitro* concentrations cannot be equated with pediatric systemic pharmacokinetics, and chronic exposure, irreversible physeal closure, donor age, and developmental-stage fidelity remain difficult to model. Standardized functional endpoints—longitudinal expansion, column organization, matrix turnover, mineralization, bone-bridge formation, and reversibility—are also lacking. Growth plate-on-a-chip systems could add perfusion, controlled gradients, and repeated exposure, but require cross-platform validation. Current growth plate organoids are promising mechanistic bridges but are not yet validated predictors of pediatric retinoid safety or clinical dose thresholds.

### 11.2 Receptor-subtype and dose-window studies

Future experiments should compare RARα-, RARβ-, and RARγ-selective antagonists in the same model, include receptor occupancy and pharmacokinetic measurements, and prespecify whether the intervention is concurrent prophylaxis, early post-injury modulation, or treatment of an established lesion. Dose-ranging should evaluate both intended effects and normal growth plate function, with stopping thresholds for impaired proliferation, abnormal hypertrophy, matrix disruption, or off-target organ toxicity.

### 11.3 Pediatric longitudinal evidence

Prospective drug-exposure cohorts are needed for children receiving systemic retinoids or other agents associated with physeal injury. Core outcomes should include standardized height velocity, sitting height, limb proportions, bone age, pubertal development, skeletal imaging when clinically indicated, bone mineral density, fractures, pain, function, and final adult height. Registries should capture dose, cumulative exposure, co-medications, nutrition, endocrine disorders, malignancy treatment, and genetic susceptibility.

### 11.4 A staged translational roadmap

A defensible development pathway would proceed from mechanism confirmation in human-derived systems to staged juvenile safety studies and indication-specific risk-benefit assessment. Any first-in-child study would require a severe condition with substantial unmet need, robust preclinical rescue, reversible pharmacology where possible, independent safety oversight, and follow-up through skeletal maturity. Adult cartilage indications cannot serve as a shortcut to pediatric growth plate use.

## 12 Conclusion

RA/RAR signaling is an essential, tightly regulated component of growth plate development. Its effects vary with dose, timing, tissue, developmental stage, and receptor subtype, while zone-specific receptor dominance in the human physis remains unresolved. Experimental antagonists have produced model-specific effects, including concurrent prevention of drug-induced closure and partial attenuation during an early post-injury window; neither establishes reversal of mature damage or pediatric efficacy. Clinical retinoid experience also shows that pathway modulation can irreversibly damage open physes. RAR antagonism should remain preclinical and hypothesis-generating until human-relevant models, receptor-specific dose windows, off-target effects, and long-term growth and reproductive safety have been adequately characterized.
