# Correction: Retinoic acid signaling in growth plate development: receptor-specific effects, antagonist evidence, and pediatric translational considerations

**DOI:** 10.3389/fendo.2026.2029390

**Published:** 2026-10-21

**Authors:** Yutong Xing, Ruiying Fan, Kaixi Wang, Xinyu Ma, Fan Xuan, Huifeng Zhang

**Affiliations:** 1 Department of Paediatrics, The Second Hospital of Hebei Medical University, Shijiazhuang, Hebei, China; 2 Hebei University of Chinese Medicine, Shijiazhuang, Hebei, China

**Keywords:** chondrocyte, endochondral ossification, epiphyseal closure, growth plate, pediatric growth, RAR antagonist

The funder “Natural Science Foundation of Hebei Province (Grant No. H2023206222)” was erroneously omitted.

The correct **Funding** statement reads:

“The author(s) declare that financial support was received for the research, authorship, and/or publication of this article. This work was supported by the 2026 Medical Science Research Project of Hebei Province, China (Grant No. 20260284) and the Natural Science Foundation of Hebei Province (Grant No. H2023206222).”

The original version of this article has been updated.

